# Dynamic Recrystallization Behavior and Processing Map of the 6082 Aluminum Alloy

**DOI:** 10.3390/ma13051042

**Published:** 2020-02-26

**Authors:** Dao-chun Hu, Lei Wang, Hong-jun Wang

**Affiliations:** 1 Engineering Technology Training Center, Nanjing Institute of Industry Technology, Nanjing 210023, China; whj_niit@163.com; 2 Jiangsu Research and Development Center of Precision Manufacturing, Nanjing 210023, China; 2018100944@niit.edu.cn; 3 School of Mechanical Engineering, Nanjing Institute of Industry Technology, Nanjing 210023, China

**Keywords:** 6082 aluminum alloy, dynamic recrystallization, processing map, hot formability

## Abstract

Multiple hot-compression tests were carried out on the 6082 aluminum (Al) alloy using a Gleeble-1500 thermal simulation testing machine. Data on flow stresses of the 6082 Al alloy at deformation temperatures of 623 to 773 K and strain rates from 0.01 to 5 s^−1^ were attained. Utilizing electron backscatter diffraction (EBSD) and a transmission electron microscope (TEM), the dynamic recrystallization behaviors of the 6082 Al alloy during hot compression in isothermal conditions were explored. With the test data, a hot-working processing map for the 6082 Al alloy (based on dynamic material modeling (DMM)) was drawn. Using the work-hardening rate, the initial critical strain causing dynamic recrystallization was determined, and an equation for the critical strain was constructed. A dynamic model for the dynamic recrystallization of the 6082 Al alloy was established using analyses and test results from the EBSD. The results showed that the safe processing zone (with a high efficiency of power dissipation) mainly corresponded to a zone with deformation temperatures of 703 to 763 K and strain rates of 0.1 to 0.3 s^−1^. The alloy was mainly subjected to continuous dynamic recrystallization in the formation of the zone. According to the hot-working processing map and an analysis of the microstructures, it is advised that the following technological parameters be selected for the 6082 Al alloy during hot-forming: a range of temperatures between 713 and 753 K and strain rates between 0.1 and 0.2 s^−1^.

## 1. Introduction

The Al–Mg–Si (aluminum–magnesium–silicon) series of wrought aluminum alloy offers excellent specific strength, workability, and corrosion resistance: it is widely applied in the aerospace and automobile industries. It is important in the realization of light-weight structures; however, due to the macrosegregation of the Mg_2_Si phase and surplus Si, the as-cast Al–Mg–Si series of wrought Al alloys contains large grains and an extremely nonuniform phase distribution; thus, its mechanical properties cannot satisfy practical service requirements [1,2]. Therefore, for the Al–Mg–Si series of wrought Al alloys, it is necessary to improve the distribution and macrosegregation of the second phase of the materials through optimization by using a thermal deformation process. In this way, we can expect to attain uniform microstructures with a fine grain size, thus greatly improving the comprehensive mechanical properties.

As a novel Al–Mg–Si series of wrought Al alloys, the 6082 Al alloy retains its high strength after heat treatment. Numerous scholars have studied the influence of heat treatment and thermal deformation conditions on the mechanical properties and microstructures of the 6082 Al alloy. Liu et al. [3] have shown that, with increasing solution temperature, the strength of the 6082 Al alloy first increases and then decreases and reaches its maximum at 793 to 813 K, while the elongation constantly increases. By conducting isothermal compression tests on 6082 Al alloy specimens subjected to solution treatment, Liu [4] found that the average grain size of the materials increases with an increasing strain rate: with increasing deformation temperature, the average grain size decreases, and mixed grains tend to disappear. Moreover, at a low-deformation temperature, the grain size of materials first increases, then decreases with increasing deformation; however, at a high deformation temperature, the grain size is unaffected by deformation. Kumar et al. [5] have investigated the influence of the deformation temperature on the precipitation of precipitated phases, microstructure evolution, mechanical properties, and the corrosion resistance of the 6082 Al alloy: they suggested that the 6082 Al alloy is more likely to undergo microstructural evolution (such as recovery and dynamic recrystallization) at a high temperature. Ma et al. [6] have experimentally analyzed the influence of technological parameters on the mechanical properties of the AA6082 Al alloy and revealed the strengthening mechanism of the Al alloy by observing and analyzing the distribution of precipitated phases. By employing rapid infrared thermal processing, Chang et al. [7] have found a large initial critical strain on the 6082 Al alloy during rapid solution treatment and a more significant precipitation strengthening effect. Thus, the mechanical properties of materials were strengthened. Cecilia et al. [8] have described the strain hardening, dynamic recovery, and static recrystallization of the 6082 Al alloy during thermal deformation by utilizing a dislocation density model. They also explained the evolution of grain size after recrystallization of the alloy. By conducting a discontinuous thermal simulation test on the static softening behavior of the A6082 Al alloy, Li et al. [9] explored the quantitative relationship between the volume fraction during recrystallization and the static softening fraction of the A6082 Al alloy. Moreover, they also established a dynamic model for static recrystallization of the material.

The microstructure of the Al alloy changes during thermoplastic forming: for favorable mechanical properties, fine and uniform grain structures are required. As one important means for the structural refinement of materials, dynamic recrystallization is used in the optimization of technological parameters for material formation and prediction and control over the microstructure. It is thought that metals (e.g., Al and Al alloys) with a high stacking fault energy are mainly subjected to dynamic recovery while barely undergoing dynamic recrystallization during thermoplastic deformation [10,11]. Additionally, it is also hard to observe fine subgrain structures in these metals through metallography; however, with the development of electron microscopy, the low-angle grain boundary (LAB) of subgrains can be observed using electron backscatter diffraction (EBSD) technology. By employing EBSD technology, Sakai et al. [12] found the LAB of the 7475 Al alloy: that is, subgrain structures formed after deformation. Moreover, they suggested that as deformation develops, the high-angle grain boundary (HAB) is eventually generated after the misalignment of the subgrain boundary reaches a critical value, thus further forming new recrystallized grains. Parvizian et al. [13] established a model for the dynamic evolution of microstructures during thermal deformation of the 6082 Al alloy. Using EBSD technology, they measured the grain and subgrain structures after thermal deformation of the 6082 Al alloy and revealed the mechanism of thermal deformation of the 6082 Al alloy based on dynamic recrystallization and allied geometric changes.

Using a hot-working processing map based on dynamic materials model(DMM), the stable deformation zone and unstable deformation zone during formation can be revealed. Moreover, the different zones shown in a hot-working processing map correspond to different deformation mechanisms as well as different microstructures and properties [14]. By utilizing a hot-working processing map and microstructural observation, the plastic deformation mechanism of materials under different deformation conditions can be analyzed and predicted. Moreover, the processing technologies used for hot-forming such materials can be optimized to allow for the best control over their microstructures and properties; therefore, a hot-working processing map based on DMM provides a means for theoretical research aimed at avoiding the generation of hot-working process defects, which saves time during process design and leads to a stable workpiece with favorable microstructures and properties.

The purpose of the present paper is to address the dynamic recrystallization behavior of the 6082 Al alloy during hot deformation. Using a thermal simulation test of isothermal conditions, transmission electron microscopy (TEM), and EBSD technology, the evolution of mechanical properties and microstructures of the 6082 Al alloy under different thermal deformation conditions was explored. On this basis, a model for dynamic recrystallization of the alloy was established, and a hot-working processing map based on DMM was obtained, which allows for a discussion of the mechanisms of dynamic recrystallization of this Al alloy. We thus provide here experimental data and a theoretical basis for designing hot-forming technologies using the 6082 Al alloy.

## 2. Experimental Procedures

The materials used in the experiment were 6082 Al alloy rods provided by Taizhou Baida Electric Appliance Co., Ltd, Taizhou, China. The composition of the 6082 Al alloy rods tested here was as follows (mass fraction, %): Si 0.95%, Fe 0.18%, Cu 0.06%, Mn 0.45%, Mg 0.65%, Cr 0.12%, Zn 0.005%, Ti 0.03%, and otherwise Al. The rods were cut into cylindrical specimens measuring Φ 8 mm × 12 mm using wire-cut electrical discharge machining (WEDM). A groove with a depth of 0.2 mm was processed at the two ends, into which lubricant was placed to reduce the effect of friction on the resulting stress state. A unidirectional thermal compression test was carried out on the 6082 Al alloy using a Gleeble-1500 thermal simulation testing machine at a heating rate of 1 °C/s, a holding time of 5 min, and a total compressive strain of 70%. During thermal compression, deformation temperatures were set to 623, 673, 723, and 773 K: strain rates were set to 0.01, 0.1, 1, and 5 s^−1^, respectively. After each test, the samples were immediately subjected to water quenching to immobilize high-temperature deformed microstructures (this allowed for easier observation of the microstructural characteristics after thermal deformation).

Utilizing EBSD and TEM, the central longitudinal sections of the deformed microstructures of the compressed specimens were studied. The EBSD specimens were first ground to 2000# using flint and fine waterproof abrasive paper, then mechanically polished until there were no scratches on the surface (using diamond polishing, with particle sizes of 3 and 0.5 μm). After polishing, the samples were ultrasonically cleaned for 5 min and then dried before electropolishing with a polishing slurry containing 90% HClO_4_ and 10% C_2_H_6_O, with a voltage of 20 V, a current of 0.3 A, and a temperature of −293 K. The microtexture of the electropolished samples was measured using a JSM-7001F/JEOL FEG-SEM (JEOL Ltd, Tokyo, Japan) with an HKL Channel 5 EBSD system attached. The microscope was operated at 30 KV, with a working distance of 18 mm, a beam current of 4 nA, and a high-resolution of 1.2 nm to obtain high-quality patterns at a high temperature. The EBSD data were acquired at a step size of 2 μm. In certain cases, a smaller step size of 0.3 μm was used to capture finer details. For texture analysis, a line scan was performed, and the scanned area included almost 2000 grains. The measured orientations, including the texture, were calculated using Channel 5 (OXFORD Instruments, Abingdon, United Kingeom). The grain boundaries were characterized as low-angle boundaries (LABs) with a misorientation range of 2–15°, as high-angle boundaries (HABs) with a misorientation greater than 15°, and as coincidence site lattice (CSL) boundaries with a specific axis–angle pair. The grain size was estimated using the area fraction method, in which the diameter of a circle equivalent in area is reported. TEM samples were mechanically thinned with emery paper and then polished and ground to below 100 μm. Afterwards, TEM specimens (Φ 3 mm) were separated by applying a puncher and then cleaned using acetone. Subsequently, by applying an MTP-II twin-jet electropolisher, the samples were thinned to less than 300 nm through liquid injection of 10% HClO_4_ and 90% CH_3_OH, with a voltage of 20 V, a current of 0.1 A, and a temperature of −293 K. In order to observe defects (such as dislocations and twins) in the microstructure more clearly, dark-field TEM imaging was selected, and the acceleration voltage was 20 kV.

## 3. Results and Discussions

### 3.1. True Stress–Strain Curve

**Figure 1:**
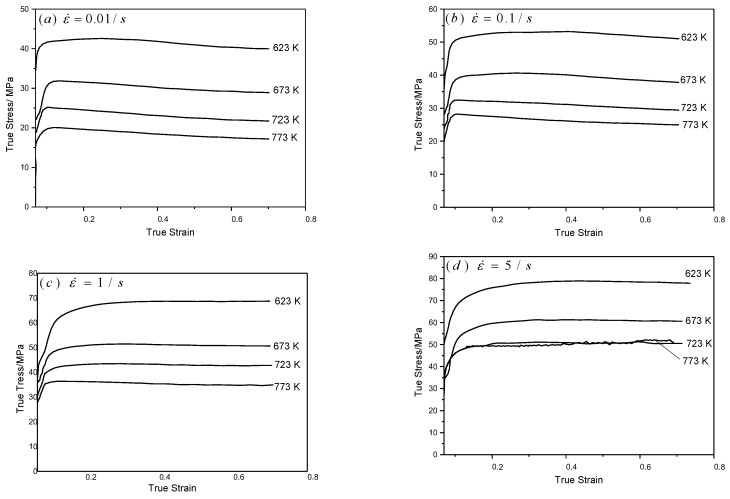
True stress–strain curves of 6082 Al alloy hot compression at different strain rates.

Figure 1 shows the stress–strain curves of the 6082 Al alloy at strain rates of 0.01 to 5 s^−1^ and deformation temperatures of 623 to 773 K. The flow stress was affected by the deformation temperature and strain rate: with increasing deformation temperature or a reduction in the strain rate, the corresponding peak stress decreased. By comparing true stress–strain curves at different strain rates, it can be seen that at low strain rates (Figure 1a,b), the Al alloy entered a steady-state flow stage with little strain (about 0.1), and the flow stress decreased thereafter: this indicated that, with a constant increase in strain, the dynamic softening effect of materials was constantly strengthened at a low strain rate, which was more significant than the work-hardening effect. At a high strain rate (Figure 1c,d), the Al alloy started to enter the steady-state flow stage after the strain exceeded 0.3. In this case, the stress remained constant, which implied that there was a certain equilibrium between the dynamic softening and work-hardening effects. Above all, with increasing temperature or a decreasing strain rate, the flow stress of the 6082 Al alloy decreased until the Al alloy entered a steady-state flow stage. In this process, the effect of strain hardening decreased while the dynamic softening effect was enhanced. Moreover, dynamic recrystallization probably occurred. The occurrence of peak stress in the true stress–strain curve shows that dynamic recrystallization occurred during thermoplastic forming; however, it was hard to judge the extent of the material deformation based on peak stress from the flow curves alone.

Sellars and Mctegart [15] have proposed a hyperbolic sine model containing activation energy *Q* for dynamic recrystallization and deformation temperature *T*. The model is used to describe the quantitative relationship between various thermodynamic parameters (e.g., the flow stress, deformation temperature, and strain rate) during high-temperature plastic deformation. To consider the effects of the strain rate and deformation temperature on dynamic recrystallization, the Zener–Hollomon factor (parameter *Z*) is introduced:

(1) $$Z=\dot{\varepsilon }\exp\left(\frac{Q}{RT}\right)$$

(2) $$\dot{\varepsilon }=A_{1}\sigma^{n_{1}}\exp(-\frac{Q}{RT})\;(\alpha \sigma \;<\;0.8)$$

(3) $$\dot{\varepsilon }=A_{2}\exp(\beta \sigma )\exp(-\frac{Q}{RT})\;(\alpha \sigma \;>\;1.8)$$

(4) $$\dot{\varepsilon }=A_{}{\left[\sinh(\alpha \sigma )\right]}^{n}\exp(-\frac{Q}{RT})\;(\mathrm{for}\;\mathrm{all}\;\mathrm{stress})$$

where, *Z*, $\dot{\varepsilon }$, *Q*, *R*, σ, and *T* refer to the Zener–Hollomon parameter, strain rate (s^−1^), activation energy (kJ/mol) for thermal deformation, molar gas constant (8.3145 J/(mol·K)), flow stress (MPa), and deformation temperature (K), respectively; *A*_1_, *A*_2_, *A*, *n*_1_, *n*, *α*, and *β* denote material constants; and $\alpha =\beta /n_{1}$.

Taking the logarithm of each side in Equations (2) and (3):

(5) $$\ln\dot{\varepsilon }=\ln A_{1}+n_{1}\ln\sigma$$

(6) $$\ln\dot{\varepsilon }=\ln A_{2}+\beta \sigma$$

**Figure 2:**
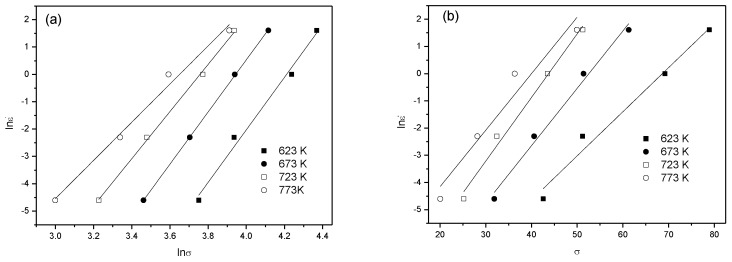
Relationship between flow stress and the strain rate at different temperatures: (**a**) $\ln\dot{\varepsilon }\sim \ln\sigma$; (**b**) $\ln\dot{\varepsilon }\sim \sigma$.

Origin software was used to obtain a best fit for the stress peak data obtained through thermal compression testing of the 6082 Al alloy, and the $\ln\dot{\varepsilon }\sim \ln\sigma$ and $\ln\dot{\varepsilon }\sim \sigma$ curves can be separately obtained, as shown in Figure 2. The average slope of the curves is *n*_1_ = 8.6877 and *β* = 0.2036, and thus $\alpha =\beta /n_{1}=0.0234$.

Accordingly, Equation (4) gives

(7) $$\ln\dot{\varepsilon }=\ln A+n\ln[\sinh(\alpha \sigma )]-\frac{Q}{RT}$$

**Figure 3:**
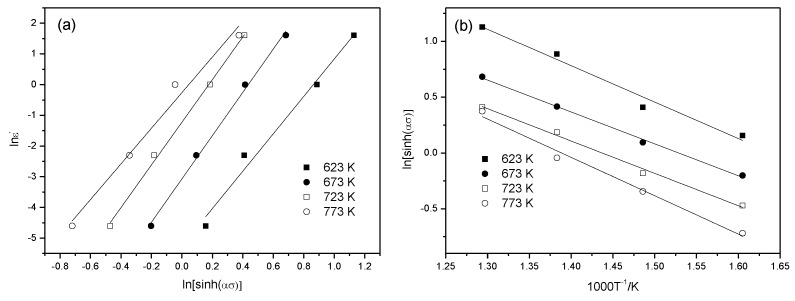
The relationship between flow stress, the strain rate, and the deformation temperature: (**a**) $\ln\dot{\varepsilon }\sim \ln[\sinh(\alpha \sigma )]$; (**b**) ln[sinh(ασ)]~1000/T.

Here, the $\ln\dot{\varepsilon }\sim \ln[\sinh(\alpha \sigma )]$ and ln[sinh(ασ)]~1000/T curves are plotted in Figure 3: the double-logarithm relationship between the hyperbolic sine of flow stress and the strain rate, as well as the relationship between the logarithm of the hyperbolic sine of flow stress and the reciprocal temperature, are quasi-linear. It is thought that the stress and strain rate during high-temperature deformation of the 6082 Al alloy satisfies the hyperbolic sine relationship in the form of an Arrhenius equation; therefore, an Arrhenius relationship in the form of a hyperbolic sine containing the activation energy *Q* for deformation can be applied to describe the flow stress on materials during high-temperature deformation, as shown in Equation (4) [16]. The partial differentiation of both sides (Equation (7)) is

(8) $$Q=R{\left\{\frac{\partial \ln\dot{\varepsilon }}{\partial \ln[\sinh(\alpha \sigma )]}\right\}}_{T}{\left\{\frac{\partial \ln[\sinh(\alpha \sigma )]}{\partial (1/T)}\right\}}_{\dot{\varepsilon }}$$

By substituting the average slopes of the curves in Figure 3a,b into Equation (8), it can be seen that *Q* = 163.5337 kJ/mol. According to Equation (8), it can be seen that the average slope of the curve in Figure 3a gives *n* = 6.4741, with the intercept of lnA−Q/(RT) at −2.4527. By substituting *Q*, R, and *T* into Equation (8), *A* = 11.9605 can be calculated. By substituting the above parameters into Equation (4), the equation for flow stress on the 6082 Al alloy at a high temperature was obtained:

(9) $$\dot{\varepsilon }=A{\left[\sinh(\alpha \sigma )\right]}^{n}\exp(-\frac{Q}{RT})=11.9605{\left[\sinh(0.0234\sigma )\right]}^{6.4741}\exp(-\frac{167.5337}{8.3145T})$$

### 3.2. Modeling of Dynamic Recrystallization

As mentioned above, during high-temperature plastic deformation of the Al alloy, it is hard to observe fine subgrain structures in the metallographs; moreover, the true stress–strain curve of the Al alloy also does not show significant peak stress. Therefore, the critical point of dynamic recrystallization cannot be clearly determined from the flow curve alone. Thus, some methods for determining the critical conditions of dynamic recrystallization have been proposed: Poliak and Jonas [17] have proposed the use of the work-hardening rate (θ=dσdε) of materials as a variable for characterizing the rate of change of flow stress with strain, which can reflect the microstructural changes in a material. Using this method, they attained accurate critical conditions for dynamic recrystallization.

**Figure 4:**
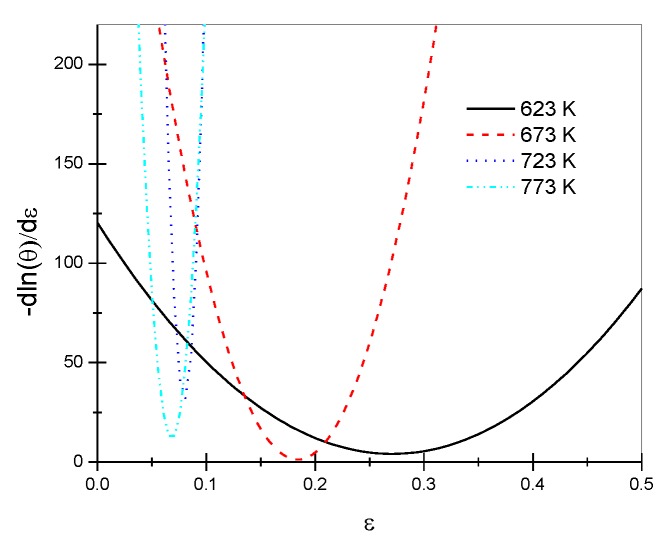
The relationship curves between −∂(lnθ)/∂ε and ε at a strain rate of 0.1 s^−1^ and different temperatures.

Utilizing Origin software, the corresponding true stress–strain curves under different deformation conditions (Figure 1) were fitted to acquire the corresponding work-hardening rate θ; moreover, the $\frac{-\partial \left(\ln\theta \right)}{\partial \varepsilon }-\varepsilon$ relationship was plotted (Figure 4) for a strain rate of 0.1 s^−1^. It can be seen that the relationship curves at different temperatures all showed a minimum point, and the corresponding strain was the critical strain causing dynamic recrystallization.

Using the above methods, the critical strains under different deformation conditions could be calculated (Table 1): the critical strain increased with an increasing strain rate or decreasing temperature. The reason for this is that, at a given deformation temperature, the greater the strain rate, the shorter the deformation time. As a result, there is no mutual timeous offset, and the dislocation density during deformation increases, with significant work-hardening tendencies exhibited. The critical strain causing dynamic recrystallization also increases. At the same strain rate, with increasing temperature, the driving force for vacancy–atom diffusion, the slip of screw dislocations, and the climb of edge dislocations increase. In this context, dynamic recrystallization is more likely to occur, so the critical strain causing dynamic recrystallization decreases [18].

**Figure 5:**
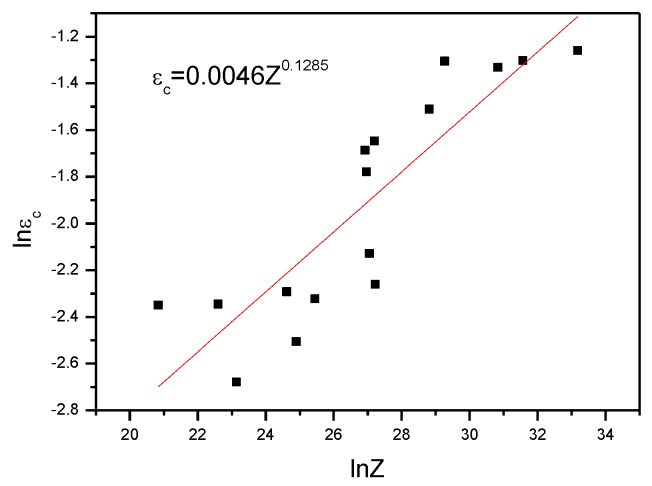
The relation between the *Z* parameter and critical strain.

Figure 5 shows the lnε−lnZ curve: the critical strain increased with increasing *Z*. The reason for this is that, when the deformation of the materials reaches critical strain, work hardening and recovery lead to the formation of dislocation substructures. Moreover, tangled dislocations, rather than clear two-dimensional (2D) grid structures, form the boundary of such substructures. The presence of high-energy-state tangled subgrain boundaries enables local zones to acquire sufficient storage energy so that the misalignment of subgrain boundaries continues to increase until an HAB is formed, leading to new dynamic recrystallization. Therefore, with an increasing strain rate and a reduction in the deformation temperature, the increasing critical strain increases the difference in storage energy across each side of the moving subgrain boundary. This guarantees that grain boundaries move rapidly to promote the coarsening of subgrains before the dislocation density at the moving boundaries reduces the initial driving force, thus realizing the growth of the nucleus during dynamic recrystallization [19]. Utilizing Origin software, linear fitting was conducted on data pertaining to the critical strain $\varepsilon_{c}$ causing dynamic recrystallization and the parameter *Z*:

(10) $$\varepsilon_{c}=0.0046Z^{0.1285}$$

The relationship between the volume fraction after dynamic recrystallization and the plastic strain and grain size after dynamic recrystallization can be separately described as follows:

(11) $$X_{DRX}=1-\exp\left\{-k{\left[\frac{\varepsilon -\varepsilon_{c}}{\varepsilon_{p}}\right]}^{m_{1}}\right\},$$

(12) dDRX=a.d0n2.ε˙n3.εn4[exp(QRT)]k1=c.Zm2,

where *X_DRX_* denotes the volume fraction after dynamic recrystallization; ε, $\varepsilon_{c}$, and $\varepsilon_{p}$ separately represent the strain, critical strain, and peak strain during high-temperature deformation; *d_DRX_* and *d*_0_ separately refer to the grain size after dynamic recrystallization and the original grain size; $\dot{\varepsilon }$, *T*, *Q*, and *R* denote the strain rate, deformation temperature, thermal activation energy, and molar gas constant, respectively; and *k*, *m*_1_, *a*, *n*_2_, *n*_3_, *n*_4_, *k*, and *m*_2_ all denote material constants.

**Figure 6:**
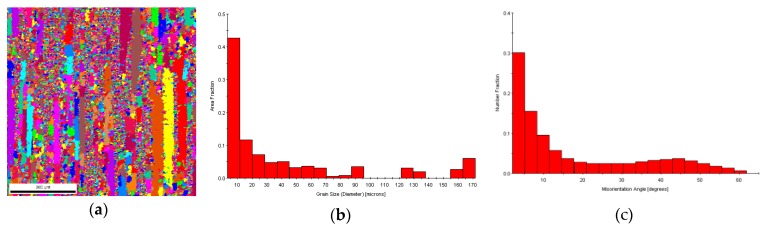
Orientation imaging microscopy(OIM) of electron backscatter diffraction (EBSD) for the 6082 Al alloy at a strain rate of 0.1 s^−1^ and a temperature of 723 K. (**a**) OIM; (**b**) grain sizes; (**c**) misorientation between grain boundaries.

Generally, the volume fraction and grain size after dynamic recrystallization can be determined by observing metallographic structures of frozen high-temperature microstructures subjected to thermal deformation; however, the volume fraction after dynamic recrystallization mainly depends on the rate of nucleation of recrystallized grains and their rate of growth. During high-temperature plastic deformation, grains subjected to dynamic recrystallization are likely to merge or grow; therefore, it is hard to observe fine grains subjected to dynamic recrystallization in microstructures even if the grain size after significant deformation is larger than that of the original grains that have not undergone deformation. As a result, it is difficult to distinguish grains subjected to dynamic recrystallization from original grains utilizing metallographic methods. By virtue of the advantages of EBSD in large-area quantitative analyses, some information on microstructures and crystallography, e.g., the size, misalignment, and distribution of recrystallized grains, can be found. The orientation mapping of the 6082 Al alloy at a deformation temperature of 723 K and a strain rate of 0.1 s^−1^ (from EBSD) is displayed in Figure 6. Different colors represent different grain orientations, and the misalignments of the subgrain boundaries and grain boundaries were generally set to 2° and 15°, respectively. The grain boundaries were characterized as very low-angle boundaries (VLABs) with a misorientation range of 2–5°, as low-angle boundaries (LABs) with a misorientation range of 5–15°, and as high-angle boundaries (HABs) with a misorientation greater than 15°. Utilizing the Recrystallized Fraction Component Function of Channel 5 software, the misalignments (Figure 6b) and percentage contents (Figure 6c) of the various grains could be attained. Correspondingly, ln *Z* = 24.9014 and *X_DRX_* = 38.60%. Using the same method, data (including the volume fraction, the grain size, and ln *Z* after dynamic recrystallization) involving different conditions could be obtained (Table 2).

By substituting the data in Table 2 into Equations (11) and (12), the dynamic model for dynamic recrystallization of the 6082 Al alloy is expressed as follows:

(13) $$X_{DRX}=1-\exp\left\{-0.330{\left[\frac{\varepsilon -\varepsilon_{c}}{\varepsilon_{p}}\right]}^{1.486}\right\},$$

(14) $$d_{DRX}=c.Z^{m_{1}}=0.0803Z^{0.256}$$

Additionally, it could be found that, at a low ln *Z* (ln *Z* = 24.9014), the volume fraction after dynamic recrystallization was large (*X_DRX_* = 38.6%), indicating that at a low strain rate and a high deformation temperature (corresponding to a low value of *Z*), dynamic recrystallization of the 6082 Al alloy was more likely to occur.

### 3.3. Processing Map of the 6082 Al Alloy

A map describing the workability of materials is called a processing map. A dynamic materials model (DMM) is established using various fundamental principles, e.g., physical system simulation, continuous mechanics, and irreversible thermodynamics. A hot-working processing map based on this model is widely used in the characterization of the hot-workability of materials and the optimization of technological parameters pertinent to hot-working [20]. In a DMM, the heating process is regarded as a system. During hot-forming, the total externally input power is expressed as $P=\sigma \dot{\varepsilon }$. The majority of the total power consumption is transformed into a visco-plastic heat induced by the plastic deformation of materials (the dissipation capacity *G*). The heat dissipated by the changes (such as dynamic recovery and dynamic recrystallization) in the microstructures during material deformation (the associated dissipation *J*) [21] can be expressed as follows:

(15) $$P=\sigma \cdot \dot{\varepsilon }=G+J=\int_{0}^{\dot{\varepsilon }}\sigma d\dot{\varepsilon }+\int_{0}^{\sigma }\dot{\varepsilon }d\sigma$$

The proportions of the two energies are determined by the sensitivity index (*m*) of the materials to the strain rate, that is,

(16) m=∂J∂G=(ε˙∂σ)(σ∂ε˙)=∂(lnσ)∂(lnε˙)

The efficiency factor (*η*) of power dissipation is introduced to characterize the dissipation of power when the microstructures of the materials change:

(17) η=JJmax=JP2=2(P−GP)=2(1−1σε˙∫0ε˙σdε˙)=2mm+1

The contour map of the power dissipation factor is drawn on a 2D plane consisting of the strain rate $\left(\dot{\varepsilon }\right)$ and the deformation temperature (T). The map describes the power dissipation conditions during microstructural evolution under deformation. Using metallographic observation, the deformation mechanisms (such as dynamic recovery and dynamic recrystallization) of microstructures due to different deformation conditions can be analyzed.

When establishing a processing map based on DMM, the instability of materials is generally judged according to the dimensionless parameter $\xi \left(\dot{\varepsilon }\right)$, which is defined in Equation (18) [22]. The contour map of the instability criterion is drawn on a 2D surface and consists of the strain rate $\left(\dot{\varepsilon }\right)$ and the deformation temperature (T), thus forming a map showing material instability. The processing map can be obtained by overlapping the map of material instability onto that showing the power dissipation. Through the processing map, the high-temperature deformation mechanism of materials in different conditions can be analyzed, and the instability zone and safe zone of materials can be determined. This method provides a theoretical and actual basis for optimizing the technological parameters of the hot-working of materials and controlling their deformed microstructures. Equation (18) is

(18) ξ(ε˙)=∂lg[m(m+1)]∂lgε˙+m.

It can be seen from Figure 2a that lgσ was linearly correlated with $\mathrm{lg}\dot{\varepsilon }$ at different temperatures; therefore, it was feasible to calculate the hot-working processing map of the 6082 Al alloy using the DMM calculation method and to determine the zone of flow instability and the safe zone from the processing map.

Experimental data pertaining to lgσ and $\mathrm{lg}\dot{\varepsilon }$ were fitted by applying a cubic spline function. Afterwards, using Equation (16), the sensitivity index (*m*) of the strain rate was calculated, while the efficiency factor *η* of power dissipation was computed using Equation (17). According to the instability criterion shown in Equation (18), $\xi \left(\dot{\varepsilon }\right)$ was obtained similarly through curve fitting by employing a cubic spline function. On the basis of DMM, the data calculated using the hot-working processing map of the 6082 Al alloy are presented in Table 3.

**Figure 7:**
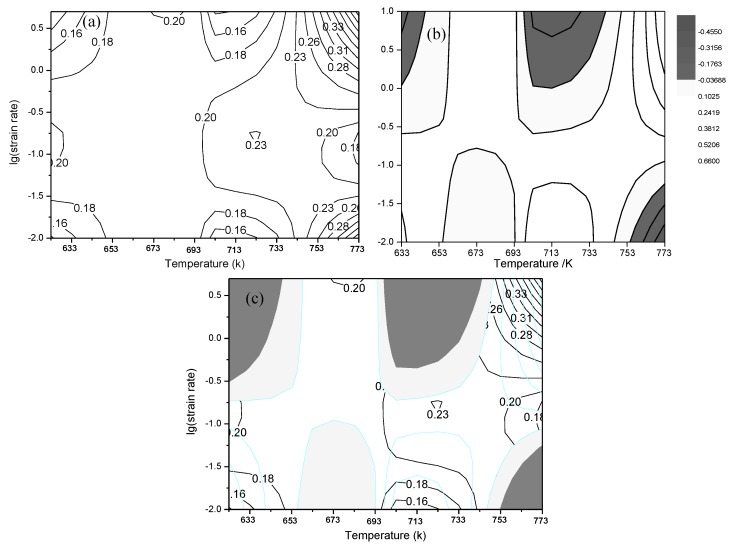
The power dissipation map (**a**), instability map (**b**), and processing map (**c**) of the 6082 Al alloy.

On the basis of the data in Table 3, maps about power dissipation and instability were separately drawn; afterwards, the map of material instability was superimposed onto the map showing power dissipation to acquire the hot-working processing map of the 6082 Al alloy (Figure 7).

As is shown in the power dissipation map (Figure 7a), three peak zones (the zones with maximum *η*) were found: a zone at 713 to 753 K, a strain rate of 0.1 to 0.2 s^−1^, and a power dissipation factor of about 0.23 (the temperature and strain rate corresponding to the peak were 723 K and 0.2 s^−1^); a zone at 743 K to 773 K, a strain rate of 0.3 to 5 s^−1^, and a power dissipation factor of about 0.23 to 0.33; and a zone at 743 K to 773 K, a strain rate of 0.01 to 0.03 s^−1^, and a power dissipation factor of about 0.23 to 0.28. The material instability map (Figure 7b) showed that the 6082 Al alloy exhibited three instability zones: a low-temperature zone with a high strain rate at 623 K to 653 K and a strain rate of 0.3 to 5 s^−1^; a medium-temperature zone with a high strain rate at 703 K to 743 K and a strain rate of 0.3 to 5 s^−1^; and a high-temperature zone with a low strain rate at 743 K to 773 K and a strain rate of 0.01 to 0.1 s^−1^.

A power dissipation map also represents a trajectory chart of microstructures, reflecting the rate of change during hot-working and deformation. Zones with a high power dissipation factor are frequently used to define the zone of best workability; however, the failure of wedge-shaped cracks also generally occurs in zones with a high power dissipation factor. Therefore, it was necessary to validate this by applying the microstructures of corresponding samples to observe the changes in microstructures in different zones of the processing map and verify the reliability of the hot-working processing map [23].

**Figure 8:**
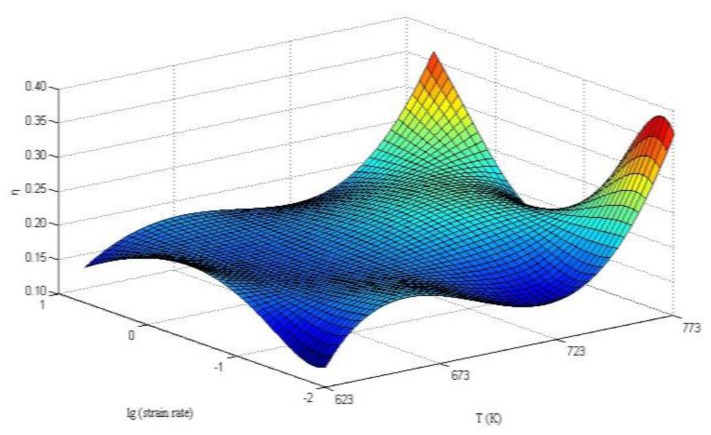
Three-dimensional representation of power dissipation as a function of strain rate and temperature of the 6082 Al alloy.

Figure 8 shows a 3D map of the efficiency of power dissipation of the 6082 Al alloy. The figure illustrates the change trends of the efficiency of power dissipation in three peak zones with different technological parameters. In two peak zones at high temperature, the efficiency of the power dissipation of the Al alloy changed significantly, indicating that the hot workability in these zones was unstable and that local flow instability probably occurred.

**Figure 9:**
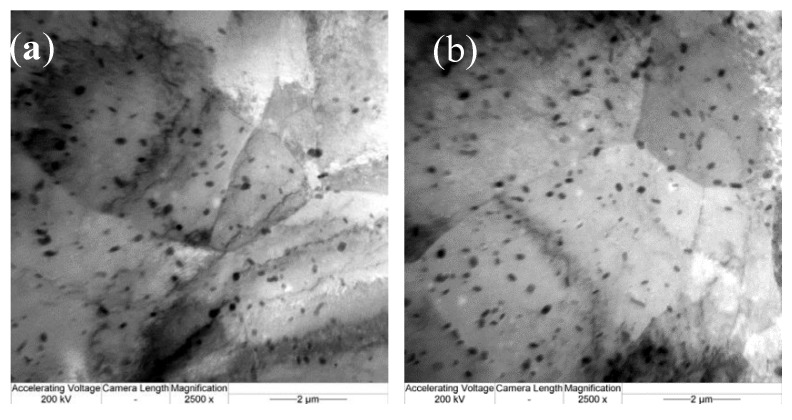

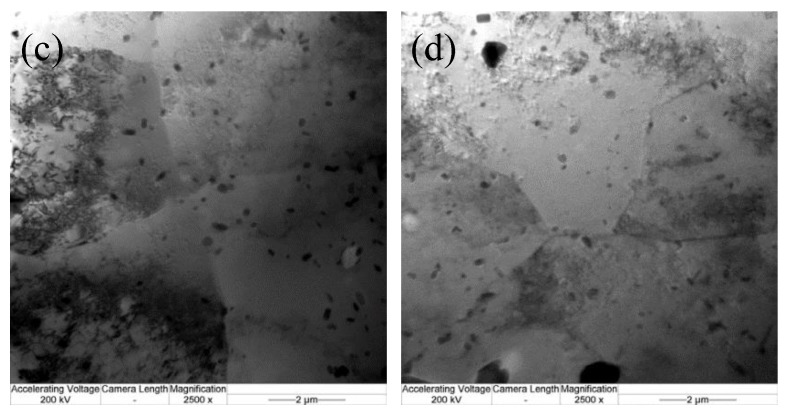
TEM micrographs of the 6082 Al alloy in different deformation conditions: (**a**) 773 K, 0.01 s^−1^; (**b**) 773 K, 5 s^−1^; (**c**) 723 K, 0.01 s^−1^; and (**d**) 723 K, 0.1 s^−1^.

At high temperatures, the deformed microstructures in the Al alloy were relatively bulky with an increase in the strain rate. As the deformation temperature increased, the degree of recovery from deformation was higher, causing the storage energy after deformation to be reduced and recrystallization nucleation to thus become more unlikely to occur. At a high strain rate, there was not enough time for the growth of dynamic recrystallization, and therefore the level of dynamic recrystallization decreased. In this case, the deformed microstructures in the Al alloy were bulky. As shown in Figure 9b, the bulky grain structures had a significant effect on the mechanical properties of the 6082 Al alloy. Thus, the 6082 Al alloy underwent negligible hot-working in the high-temperature zone at a high strain rate. In the high-temperature zone with a low strain rate, on the one hand, the deformation temperature was high, so grains grew rapidly; on the other hand, new recrystallized grains were found at the grain boundary, so the grain structures exhibited significant heterogeneity (Figure 9a). As a result, the nonuniform deformation of the grain boundary was likely to have occurred in the deformation process, triggering flow instability. Contour lines were sparse in the peak zone of the efficiency of power dissipation at 703 K to 743 K and at strain rates of 0.1 to 0.2 s^−1^. The contour lines in the peak zone are sparse, and the distribution of contour lines is uniform while maintaining a high efficiency factor of power dissipation, an shown in Figure 7a. The TEM image of the samples at 723 K shows that at a strain rate of 0.01 s^−1^, the grains in the Al alloy were bulky, and many tangled dislocations were found (Figure 9c). A possible reason for this was that the degree of recovery was low at a low strain rate, so it was difficult to prevent increasing dislocation density and grain crushing in the deformation process through recovery. Under these conditions, dynamic recrystallization had not yet occurred, so the grains remained bulky. With an increasing strain rate, the number of tangled dislocations gradually decreased (Figure 9d), and the substructures became clearer.

**Figure 10:**
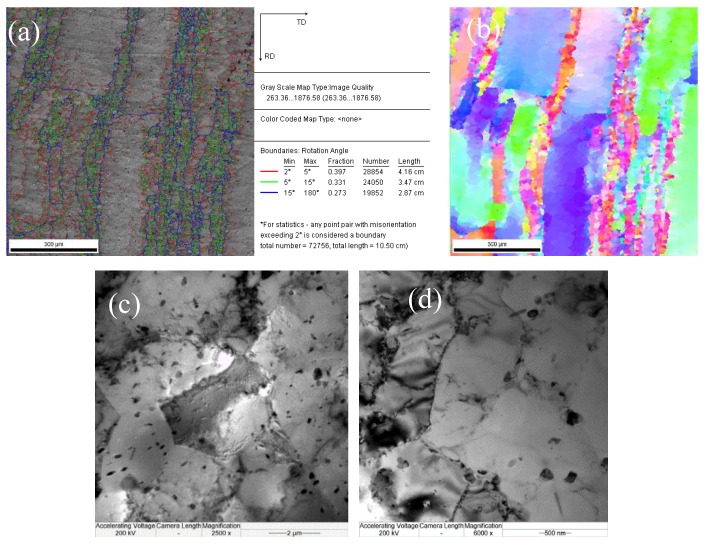
Microstructures of dynamic recrystallization softening of the 6082 Al alloy at 723 K, 0.1 s−1: (**a**) the distribution of LAGBs and HAGBs; (**b**) a grain orientation map; (**c**,**d**) and a TEM micrograph of the substructures.

At a strain rate of 0.1 s^−1^, an apparent subgrain boundary appeared, with significant recrystallization characteristics (Figure 10). A chart showing the grain angles (Figure 10a) revealed that the grain boundary represented by the blue lines was misaligned, and some fine new grains appeared (Figure 10b). The corresponding TEM images (Figure 10c) show that the dislocation in the grains disappeared, and therefore the dislocation density was significantly reduced, implying that dynamic recrystallization occurred. The microstructures presented in Figure 10 indicate a higher fraction of VLABs than LABs for all deformation levels. VLABs can form either during deformation or annealing. The geometrically necessary dislocations generated during deformation can manifest in the form of VLABs to maintain strain compatibility. On the other hand, dislocation annihilation during recovery can also give rise to the formation of VLABs. In the case of deformation, increases in the fraction of VLABs should be associated with a concomitant increase in LABs. However, an analysis of the EBSD scans clearly revealed that increases in the fraction of LABs were not as significant as those of the VLABs. Therefore, it could be inferred that the large increase in VLABs was mostly associated with recovery. The microstructural features also indicated that there was a consistent increase in the fraction of HABs, which is a clear indication of the occurrence and progress of dynamic recrystallization.

During plastic deformation of Al alloys at high temperatures, the stacking fault energy in the Al alloy is high, so the extended dislocations are narrow and are likely to become bundled during deformation. As a result, the climbing of edge dislocations and the cross-slipping of screw dislocations are likely to be transferred between slip surfaces. In this context, unlike dislocations that are mutually offset and disappear, the distribution of dislocations is also changed and forms a closed cell wall to cut originally intact grains into many zones with a low dislocation density [24]. As deformation continues, cell walls of dislocations are subjected to polygonization to form regular boundaries, thus generating an LAB (Figure 10b, represented using red lines), with subgrain structures (Figure 10d) forming after deformation. Additionally, the dislocation generated in subsequent deformation continues to move and converges at the LAB, thus causing increasing misalignment of the subgrain boundary. With sufficient deformation, the misalignment of the subgrain boundary reaches its critical value, and eventually subgrain boundaries evolve into an HAB, thus forming new recrystallized grains [25]. The recrystallization process (without the participation of a nucleation–growth mechanism) is completed as deformation accumulates, indicating the evolution of microstructural features during continuous dynamic recrystallization of the alloy [26].

Dynamic recrystallization can eliminate many of the dislocations generated in the forming process of materials (through grain refinement), and it also maintains a certain dynamic equilibrium. Additionally, the efficiency of power dissipation is high within the zone, and therefore microstructures of such materials are improved, thus guaranteeing their stress stability and favorable workability during hot-working. Therefore, A zone with dynamic recrystallization should be selected preferentially when determining technological parameters according to the hot-working processing map [17,18].

On the basis of the above analyses of the hot-working processing map and microstructural observations, three peak zones with a high efficiency of power dissipation should be selected preferentially when selecting the technological parameters for the hot-working of the 6082 Al alloy. Moreover, during a deformation process at high temperatures, recrystallized grains and original grains are likely to coarsen, thus impairing the material properties; therefore, during the formation of the 6082 Al alloy through hot-working, the temperature should be between 713 and 723 K, and the strain rate should be between 0.1 and 0.2 s^−1^.

## 4. Conclusions

(1) Using the work-hardening rate, the initial critical strain causing dynamic recrystallization was determined, and the quantitative relationship between critical strain and the *Z* parameter was established. Furthermore, the equation for the critical strain causing dynamic recrystallization was obtained: $\varepsilon_{c}=0.0046Z^{0.1285}$. On the basis of the results of the EBSD analysis and the test data, a model ($X_{DRX}=1-\exp\left\{-0.330{\left[\frac{\varepsilon -\varepsilon_{c}}{\varepsilon_{p}}\right]}^{1.486}\right\}$) for the volume fraction of dynamic recrystallization of the 6082 Al alloy and a model ($d_{DRX}=c.Z^{m_{1}}=0.0803Z^{0.256}$) for grain size were separately obtained;
(2) During the thermoplastic deformation process of the 6082 Al alloy, a subgrain boundary was generated in the vicinity of the original grain boundary, and the migration of the subgrain boundary triggered the coarsening of the subgrain boundary. As a result, the LAB evolved into an HAB, and new recrystallized grains were found. The process mainly involved the continuous dynamic recrystallization of the alloy;
(3) There were three peak zones with a high efficiency of power dissipation (seen in the hot-working processing map of the 6082 Al alloy), which corresponded to a safe processing zone, a processing zone with potential risk, and a stress instability zone. The hot-working processing map and the analysis of the microstructures showed that, within the deformation zone at 713 to 753 K and at strain rates of 0.1 to 0.2 s^−1^, the contour lines were sparse and uniformly distributed, while a high efficiency of power dissipation was maintained. Within the zone, the 6082 Al alloy was subjected to dynamic recrystallization (with an aligned grain orientation and arrangement), thus realizing effective hot-working of the alloy.

## Figures and Tables

**Table 1:** Critical strain under different deformation conditions.

	Strain Rate (s^−1^)	Temperature (K)			
		623	673	723	773
Critical strain	0.01	0.168850	0.101060	0.095826	0.095415
	0.1	0.271242	0.185107	0.081699	0.068627
	1	0.271989	0.094398	0.192717	0.098039
	5	0.284080	0.264006	0.220822	0.119048

**Table 2:** Experimental data involving different deformation conditions.

Temperature (K)	Strain Rate (s^−1^)	The Volume Fraction of Dynamic Recrystallization (%)	Grain Size (μm)	ln *Z*
623	0.1	25.7	87.73	29.2680
723	0.1	38.6	40.72	24.9014
723	5	27.3	222.40	28.8134
773	0.1	33.3	55.16	25.1418
773	5	13.2	312.20	27.0538

**Table 3:** Values of the 6082 Al alloy processing map.

Temperature (K)	Strain Rate (s^−1^)	m	η	$\xi \left(\dot{\varepsilon }\right)$
623	0.01	0.0676	0.1266	0.3828
	0.1	0.1162	0.2082	0.2161
	1	0.1024	0.1858	−0.1132
	5	0.0557	0.1055	−0.4399
673	0.01	0.1110	0.1998	0.0523
	0.1	0.1017	0.1846	0.0928
	1	0.1050	0.1900	0.1399
	5	0.1148	0.2059	0.1803
723	0.01	0.0833	0.1538	0.3382
	0.1	0.1273	0.2258	0.2018
	1	0.1185	0.2119	−0.0162
	5	0.0810	0.1499	−0.2170
773	0.01	0.2294	0.3732	−0.4511
	0.1	0.0976	0.1778	0.0802
	1	0.1542	0.2672	0.4705
	5	0.3056	0.4682	0.6596
